# The Association of Tobacco Smoking and Level of Apoptosis in the Long Head of the Biceps Chronic Tendinopathy—An Immunohistochemical Study

**DOI:** 10.3390/jcm13030684

**Published:** 2024-01-24

**Authors:** Łukasz M. Jaworski, Jan Zabrzyński, Peter J. Millett, Marco-Christopher Rupp, Filippo Familiari, Gazi Huri, Paulina Antosik, Michał Błachowski, Michał Wiciński, Maciej Gagat

**Affiliations:** 1 Department of Orthopaedics and Traumatology, Faculty of Medicine, Collegium Medicum in Bydgoszcz, Nicolaus Copernicus University in Toruń, 85-092 Bydgoszcz, Poland; lukaszmjaworski@gmail.com (Ł.M.J.); blachowskiort@gmail.com (M.B.); 2 The Steadman Clinic, Vail, CO 81657, USA; drmillett@thesteadmanclinic.com; 3 Department of Orthopedic Sports Medicine, Technical University of Munich, 81675 Munich, Germany; marco.rupp@tum.de; 4 Department of Orthopaedic and Trauma Surgery, Magna Graecia University, 88100 Catanzaro, Italy; filippofamiliari@unicz.it; 5 Research Center on Musculoskeletal Health, MusculoSkeletalHealth@UMG, “Magna Græcia” University, 88100 Catanzaro, Italy; 6 Orthopaedics and Traumatology Department, Hacettepe Universitesi, 06100 Ankara, Turkey; gazihuri@yahoo.com; 7 Department of Clinical Pathomorphology, Collegium Medicum in Bydgoszcz, Nicolaus Copernicus University in Toruń, 85-092 Bydgoszcz, Poland; paulinantosik@gmail.com; 8 Department of Pharmacology and Therapeutics, Faculty of Medicine, Collegium Medicum in Bydgoszcz, Nicolaus Copernicus University in Toruń, 85-092 Bydgoszcz, Poland; wicinski4@wp.pl; 9 Department of Histology and Embryology, Faculty of Medicine, Collegium Medicum in Bydgoszcz, Nicolaus Copernicus University in Toruń, 85-092 Bydgoszcz, Poland; mgagat@cm.umk.pl; 10 Faculty of Medicine, Mazovian Academy in Płock, 09-402 Płock, Poland

**Keywords:** apoptosis, tendon, tendinopathy, smoking, tobacco smoke

## Abstract

Background: The substances present in cigarette smoke have a negative impact on cellular integrity and metabolism, can reduce blood flow to tissues, and can disrupt collagen synthesis. Ultimately this can lead to cell death, which clinically may result in impaired tendon healing and the onset of chronic tendinopathy. Within the shoulder, the exact association between the extent of apoptosis in the long head of the biceps (LHB) tendon and harmful factors like cigarette smoke remains unclear. Objectives: The purpose of this study was to investigate the connection between smoking, the degree of apoptosis in LHB tendinopathy, and the long-term outcomes of surgical treatment. Design: Observational, retrospective study. Methods: This study included 22 consecutive patients who had undergone arthroscopic biceps tenodesis or tenotomy for symptomatic LHB tendinopathy with or without concomitant rotator cuff tears (RCT). The intra-articular LHB tendon remnants were histologically examined by measuring the level of expression of apoptotic cell markers such as BCL2, cleaved caspase 3, and p53. Pre- and postoperative clinical outcomes were analyzed by collecting patient report outcome measures such as the American Shoulder and Elbow Surgeons (ASES) score and the Visual Analogue Scale (VAS) for pain. Results: The smokers group had a mean pack-year history of 13.12 (SD = 9.94), mean number of cigarettes per day of 14.77 (SD = 4.64), and a mean smoking duration of 16.38 (SD = 10.1) years. Among the smoking indexes, the number of cigarettes per day showed a positive correlation with Snyder classification (*p* = 0.0459, rho = 0.3682). Non-smokers and smokers did not show a statistically significant difference in the expression indexes of BCL2, cleaved caspase 3, or p53 (*p* = 0.4216, *p* = 0.5449, *p* = 0.5613, respectively). However, the cleaved caspase 3 expression index showed a negative correlation with the severity of rotator cuff lesions in the total population (*p* = 0.0193, rho = −0.4651). Conclusions: While apoptotic processes in the LHB tendon were observed, no significant association was found between tobacco smoking, the extent of apoptosis, and clinical outcomes. However, the expression of the apoptotic marker cleaved caspase 3 correlated with the severity of rotator cuff pathology. Furthermore, active smoker status was associated with worse clinical outcomes in terms of pain following LHB tenodesis or tenotomy.

## 1. Introduction

Tobacco smoking is known to have a harmful effect on various human systems, including the musculoskeletal system [1,2,3]. Cigarette smoke contains over 4000 harmful substances, such as nicotine, aromatic hydrocarbons, carbon monoxide, nitrosamines, hydrogen cyanide, and aldehydes [4,5]. The impact of tobacco smoke on tendinous tissue is particularly damaging, as it impairs tenocyte metabolism, reduces blood flow to tissues, disrupts collagen synthesis, and accelerates tendinopathy [4,6,7]. In previous studies, correlations between smoking and the extent of degeneration of tendinous tissue in the long head of the biceps tendon (LHB) in tendinopathy, the severity of rotator cuff injury, and American Shoulder and Elbow Surgeons (ASES) score have been demonstrated [4,8,9].

Tendinopathy is a chronic tendon disorder caused by impaired regeneration, mainly characterized by the disruption of collagen bundles’ architecture, the accumulation of proteoglycans in the extracellular matrix (ECM), tenocyte transformation, vascular bed expansion, and altered cell–matrix interactions [10]. The extensive pathological alterations in the ECM, characterized by a highly disorganized microstructure of the tendon and subsequent altered cell–matrix interactions, are believed to induce the process of apoptotic cell death. However, the role of apoptosis in tendinopathy is still not well understood. In the last decade, tenocyte apoptosis has been identified as an important factor in the tendinopathy process, but the exact pathomechanism of tendinopathy remains unknown and is considered to be multifactorial [11,12,13,14,15]. Specific to the LHB, tendinopathy is typically associated with repetitive, chronic traction and friction forces and is commonly associated with rotator cuff tears (RCTs) [16,17,18].

However, to date, it remains unknown whether harmful factors such as tobacco smoking are associated with the extent of the apoptotic process in the setting of tendinopathy of the LHB, and there remains a scarcity of evidence regarding the repercussions of these apoptotic changes on the clinical outcomes. While there are studies evaluating the apoptosis process in rotator cuff pathology, smoking, and clinical outcomes, evidence on the histological and clinical effects of tobacco consumption on the adjacent LHB tendon is rather limited [7,11,19].

Thus, the aim of this study was to investigate the association between smoking, the extent of the apoptotic process in LHB tendinopathy, and long-term surgical results. We hypothesized that there would be abundant evidence of apoptotic processes in the setting of LHB tendinopathy, which would be similar to the changes found in RCTs, and that tobacco consumption would be associated with poor clinical outcomes.

## 2. Material and Methods

This study was approved by the institutional Bioethics Committee (approval number KB 598/2016), including consecutive patients who underwent shoulder arthroscopic surgery for symptomatic LHB tendinopathy.

Patients diagnosed with an LHB tendinopathy that were treated by arthroscopic LHB tenodesis and tenotomy were assessed for eligibility. The inclusion criteria for the study were as follows: patients who did not respond to conservative treatment with a minimum of three months of physiotherapy and were aged over 18 years. Exclusion criteria included a history of systemic inflammatory or rheumatic diseases, previous shoulder surgical treatment, traumatic SLAP injuries, and corticosteroid injections in the year prior to surgery.

### 2.1. Preoperative Assessment

The diagnosis of LHB tendinopathy relied both on clinical examination, including the tenderness over bicipital groove test, as well as imaging, such as sonographic examination and non-contrast magnetic resonance scans (MRI) of the shoulder. Patient demographic data, smoking status, and preoperative American Shoulder and Elbow Surgeons (ASES) score were recorded. The patient population was categorized as smokers or non-smokers (those who had never used any nicotine supplement, such as nicotine gum or patch, oral snuff/moist snuff, cigars, or cigarettes). In addition, dose-dependent and time-dependent data about smoking habits were collected, including the period of cigarette smoking (smoking years), the mean number of cigarettes smoked per day, and pack-years index (one pack contains 20 cigarettes in our country). The surgeons were blinded to the smoking status of the patients.

### 2.2. Surgical Technique

All patients underwent shoulder arthroscopy (using a standard 30° arthroscope from Smith & Nephew, Memphis, TN, USA), which was performed with the patient in the beach chair position. A standard posterior portal was used, along with any additional necessary working portals. During the surgery, the intracapsular portion of the LHB tendon was examined for the presence of tears, starting from its insertion on the superior margin of the labrum, until its entrance into the bicipital groove. The condition of the biceps pulley, stability of the LHB tendon in the groove, and the extracapsular part of the tendon were also inspected by pulling the tendon with a probe inside the joint. The Lafosse classification was used to classify LHB tears into three grades: grade 0 (normal tendon), grade I (minor lesion), and grade II (major lesion).

All patients underwent either biceps tenodesis or tenotomy, depending on the surgeon’s decision, which was based on clinical data; specifically, for tenodesis, patients who did not accept a possible Popeye deformity were qualified, as well as tenotomy patients who were older than 50 years and who accepted a possible arm deformity [20]. For the tenodesis procedure, the bicipital groove was prepared with a curette to encourage bone-tendon healing, and the suture anchor (4.5 mm double-loaded suture anchor, Twinfix, Warrington, UK; Smith & Nephew, Memphis, TN, USA) was fixed in the center of the bicipital groove. Sutures were passed through the LHBT and tied; afterwards, the tendon was cut above the suture anchor. Subsequently, the LHBT was released near its insertion at the superior labrum, and the biceps stump was removed from the shoulder using a grasping tool. For tenotomy, the LHBT was cut near its insertion to the labral complex; subsequently, the superior labrum was debrided. This technique provides an extensive biceps stump, which results in its entrapment in the area of the biceps pulley. The intraarticular portion of the LHB tendon was harvested and examined histopathologically. Concurrent rotator cuff pathology was classified according to the Snyder classification and treated accordingly [21,22].

The rehabilitation protocol after shoulder arthroscopy varied according to the surgical procedures performed and consisted of a six-week physiotherapy program. The rehabilitation protocol after tenotomy included wearing a sling for three weeks, with early passive range of motion exercises and active shoulder range of motion exercises. Active flexion of the elbow was allowed as of the third week post-surgery. On the other hand, the rehabilitation protocol after the tenodesis procedure included wearing a shoulder orthosis (medi SLK 90, Gliwice, Poland) for five weeks, along with passive shoulder range of motion exercises, followed by active exercise starting from the fourth to fifth week. Active elbow flexion and forearm supination against resistance was allowed from the eighth week. In the setting of a rotator cuff repair (RCR), a shoulder abduction orthosis was recommended.

### 2.3. Histopathological Assessment

The biceps tendon remnants from the intraarticular parts were fixed in 10% buffered formalin that was fresh and sterile. After 24 h, they were dehydrated, paraffin-embedded, and prepared for histologic evaluation using hematoxylin and eosin (H&E) staining. The 5 μm sections were examined under a light microscope (Olympus BX46, Tokyo, Japan) by two experienced observers who were blinded to the sample identity. For immunohistochemical staining, the sections were deparaffinized, rehydrated, and treated to block endogenous peroxidase with 3% hydrogen peroxide. The staining was performed using BenchMark Ultra (Ventana Medical Systems, Oro Valley, AZ, USA; Roche, Basel, Switzerland) as per the manufacturer’s instructions. The apoptotic response in tendon samples was assessed by examining the density of apoptotic cells, specifically by assessing the presence of markers such as cleaved caspase 3, p53, and BCL2. The number of positive cells was counted per entire specimen, and a scoring system was established to allow for quantification and classification of the apoptotic response in the tendon samples. In this scoring system, points were assigned depending of the number of positive cells (0 points for no positive cells, 1 point for fewer than 10 positive cells per specimen—a moderate reaction, and 2 points for more than 10 cells per specimen—an abundant reaction). Two experienced observers (P.A. and M.G.) who were blinded to the sample identity evaluated the apoptotic response and immunohistochemical evaluation in the samples.

### 2.4. Clinical Evaluation

At final follow-up, the clinical assessment included a physical examination to assess functional outcomes, as well as assessment of the American Shoulder and Elbow Surgeons (ASES) score, the Visual Analog Scale (VAS) for pain, and reoperation rate.

### 2.5. Ethics Statement

The study was performed following the Declaration of Helsinki for experiments involving humans. All patients provided written informed consent before participating in the study.

### 2.6. Statistical Analysis

Descriptive statistics were used to summarize categorical and continuous variables, with categorical variables reported as counts and percentages, and continuous variables reported as mean ± standard deviation. The distributions of variables were assessed for normality using the Shapiro–Wilk test. Correlations between the parameters studied were assessed using the Spearman rho correlation coefficient. Intergroup comparisons were performed using non-parametric tests such as the Mann–Whitney U test and analysis of variance. A *p*-value of less than 0.05 was considered statistically significant. All statistical analyses were performed using Graphpad Prism software (GraphPad 8.0.1 Software, Dotmatics, Bishop’s Stortford, UK).

## 3. Results

**Figure 1:**
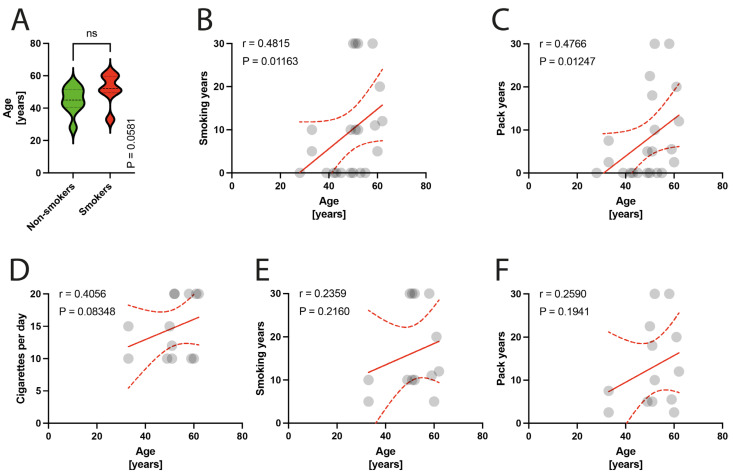
(**A**) Comparison of age in smokers/non-smokers group. (**B**–**D**) Correlation between the age and smoking years, pack-years index, and cigarettes per day in the total population. (**E**,**F**) Correlation between the age and smoking years, pack-years index in the smokers group. Circles represent values; red lines represent simple linear regressions, dotted lines represent 95% confidence intervals, ‘ns’ indicates lack of statistical significance.

Among the 25 patients included, 3 patients were excluded due to technical issues with the processing of material for microscopic examination. The remaining 22 patients were included in the final analysis. The participants were divided into two groups: smokers (13 patients) and non-smokers (9 patients). The smokers group had a mean pack-year history of 13.12 (SD = 9.94), mean number of cigarettes per day of 14.77 (SD = 4.64), and a mean smoking duration of 16.38 (SD = 10.1) years. The mean age in the studied population was 48.86 years; specifically, in the smokers group it was 44.8 years (range: 28–55 years), while in the non-smokers group it was 51.61 years (range: 33–62 years) (*p* = 0.058) (Figure 1A). The smoking parameters did not show any correlation with age in the entire studied group (Figure 1B–D) or the isolated group of smokers (Figure 1E,F).

### 3.1. Surgical technique

Within the patient population, the LHB tendon lesions could be graded as Lafosse type II lesions in 100% of the cases (*n* = 22). LHB tenodesis was performed in 18 patients, and tenotomy in 4 patients. Additionally, all of the patients underwent concomitant procedures, including RCR (C-2 according to Snyder classification) in six patients and massive RCR (C-4 according to Snyder classification) in nine patients. Seven patients had subacromial impingement with RC tendinopathy (B-1 and B-2 changes in the RC according to Snyder classification) and underwent acromion-clavicular joint (ACJ) joint resection, glenohumeral joint debridement, subacromial decompression, and coracohumeral ligament resection.

**Figure 2:**
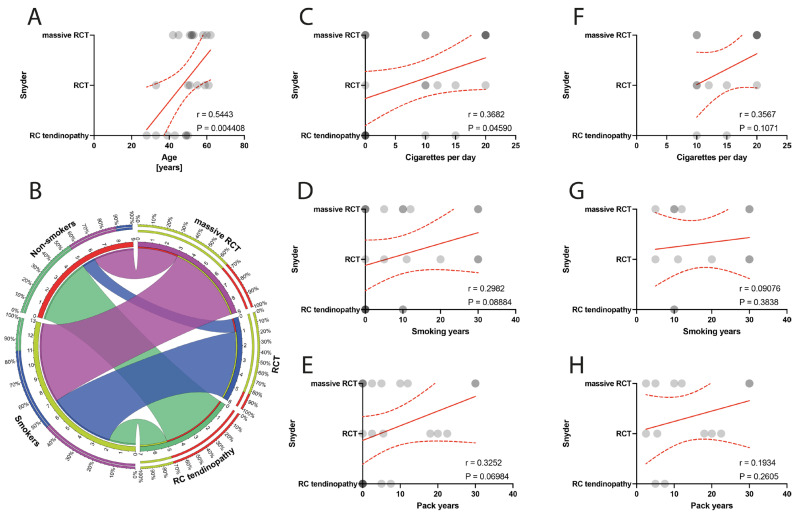
(**A**) Correlation between the Snyder classification and the age of patients. (**B**) Comparison of smoking and alterations in Snyder classification. (**C**–**E**) Correlation between the Snyder classification and smoking years, pack-years index, and cigarettes per day in the total population. (**F**–**H**) Correlation between the Snyder classification and smoking years, pack-years index, and cigarettes per day in the smokers population. Colors represent distinct groups of patients, while ribbons connecting various regions of the plot signify relationships between different data elements. Circles represent values; red lines represent simple linear regressions, dotted lines represent 95% confidence intervals.

The grade of macroscopic RC tendon lesions according to Snyder classification positively correlated with the age of patients (*p* = 0.0044) (Figure 2A). Moreover, the number of cigarettes per day positively correlated with RC defect grade according to the Snyder classification (*p* = 0.0459) (Figure 2B,C). Specifically, within the smoker group, there was no significant correlations of smoking indexes with the RC defect state as classified by the Snyder classification (Figure 2F–H).

### 3.2. Clinical Outcomes

**Figure 3:**
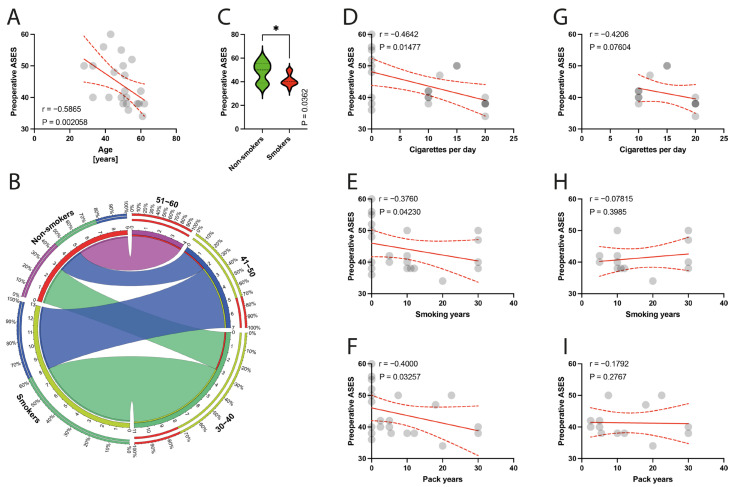
(**A**) Correlation between the preoperative ASES and the age of patients. (**B**) Comparison of smoking and age variable (30–60 years). (**C**) Comparison between smokers/non-smokers and preoperative ASES. (**D**–**F**) Correlation between the preoperative ASES and smoking years, pack-years index, and cigarettes per day in the total population. (**G**–**I**) Correlation between the preoperative ASES and smoking years, pack-years index, and cigarettes per day in the smokers population. Colors represent distinct groups of patients, while ribbons connecting various regions of the plot signify relationships between different data elements. Circles represent values; red lines represent simple linear regressions, dotted lines represent 95% confidence intervals, ‘*’ represents *p* < 0.05.

**Figure 4:**
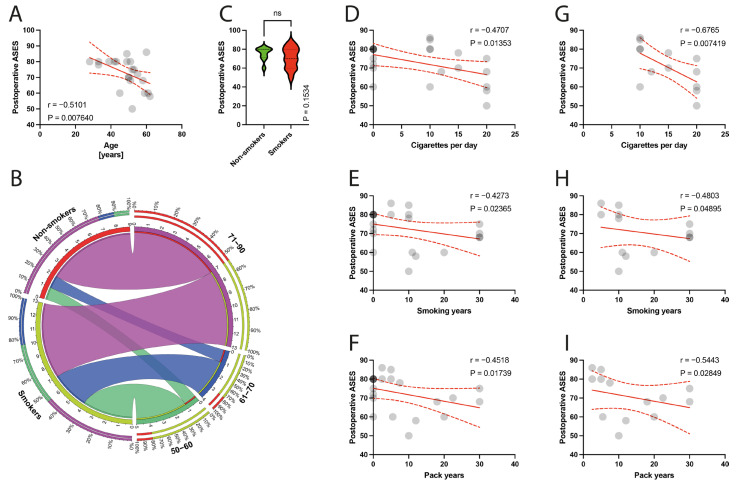
(**A**) Correlation between the postoperative ASES and the age of patients. (**B**) Comparison of smoking and age variable (50–90 years). (**C**) Comparison between smokers/non-smokers and postoperative ASES. (**D**–**F**) Correlation between the postoperative ASES and smoking years, pack-years index, and cigarettes per day in the total population. (**G**–**I**) Correlation between the postoperative ASES and smoking years, pack-years index, and cigarettes per day in the smokers population. Colors represent distinct groups of patients, while ribbons connecting various regions of the plot signify relationships between different data elements. Circles represent values; red lines represent simple linear regressions, dotted lines represent 95% confidence intervals. ‘ns’ indicates lack of statistical significance.

The average follow-up period after surgery was 40.91 months, ranging from 24 to 65 months, and there were no case of reoperations during this time. The mean preoperative ASES score in the studied group was 44.18 (SD = 7.34), and specifically in the non-smokers group, the preoperative ASES score was higher than in the smoking group (48.33; SD = 8.54 versus 41.31; SD = 4.88) (Figure 3). The mean postoperative ASES score in the studied group was 72.50 (SD = 9.79; specifically, in the non-smoking group 75.22, SD = 6.92; and in the smoking group 70.62, SD = 11.24). (Figure 4). The preoperative VAS score was mean 7.52 (SD = 1.02); for smokers it was 7.38, SD = 1.12, and for non-smokers it was 7.83, SD = 0.75. There was a statistically significant improvement in the ASES between preoperative and postoperative scores in entire population (*p* < 0.0001), as well in the smoking and non-smoking populations (*p* < 0.0001). Moreover, the surgical technique of biceps management did not bias the clinical outcomes measured using the ASES score (mean 69.6, SD = 12.15 for tenotomy; and mean 73.17, SD = 9.47 for tenodesis technique, *p* = 0.6355).

The preoperative ASES score was negatively correlated with age (*p* = 0.0021) (Figure 3A) and with smoking parameters, such as the number of cigarettes per day (*p* = 0.0148) (Figure 3D), smoking years (*p* = 0.0423) (Figure 3E), and pack years (*p* = 0.0326) (Figure 3F). Within the isolated smokers group, there were no such significant correlations (Figure 3G–I).

The postoperative ASES score was negatively correlated with the age of patients (*p* = 0.0076) (Figure 4A). There were statistically significant negative correlations between the postoperative ASES score and smoking parameters, including the number of cigarettes per day (*p* = 0.0135, rho = −0.4707) (Figure 4D), smoking years (*p* = 0.0236, rho = −0.4273) (Figure 4E), and pack years index (*p* = 0.0174, rho = −0.4518) (Figure 4F), in the total population. Within the smokers group, a significant correlation with the number of cigarettes per day (*p* = 0.0074, rho = −0.6765) (Figure 4G), smoking years (*p* = 0.0489, rho = −0.4803) (Figure 4H), and pack years index (*p* = 0.0285, rho = −0.5443) (Figure 4I) was found.

**Figure 5:**
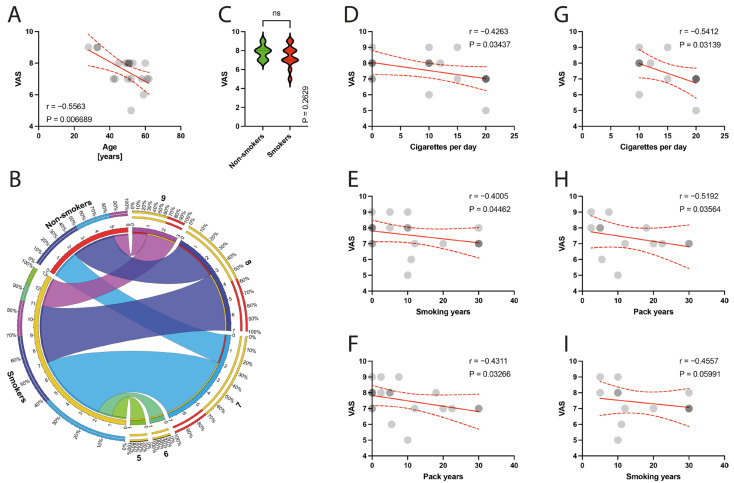
(**A**) Correlation between the VAS and the age of patients. (**B**) Comparison of smoking and VAS variable. (**C**) Comparison between smokers/non-smokers and VAS score. (**D**–**F**) Correlation between the VAS and smoking years, pack-years index, and cigarettes per day in the total population. (**G**–**I**) Correlation between the VAS and smoking years, pack-years index, and cigarettes per day in the smokers population. Colors represent distinct groups of patients, while ribbons connecting various regions of the plot signify relationships between different data elements. Circles represent values; red lines represent simple linear regressions, dotted lines represent 95% confidence intervals. ‘ns’ indicates lack of statistical significance.

The preoperative VAS score was negatively correlated with the mean age of patients (*p* = 0.0067) (Figure 5A), and there was no significant difference between non-smokers and smokers in terms of VAS scores (*p* = 0.2629) (Figure 5C). Smoking parameters were negatively correlated with the VAS score, including the number of cigarettes per day (*p* = 0.0344) (Figure 5D), smoking years (*p* = 0.0446) (Figure 5E), and pack years (*p* = 0.0327) (Figure 5F), in the total population. Within the smoker groups, a significant correlation with the number of cigarettes per day (*p* = 0.0314) (Figure 5G), smoking years (*p* = 0.0356) (Figure 5H), and VAS score was demonstrated.

### 3.3. Immunohistochemical Examination

**Figure 6:**
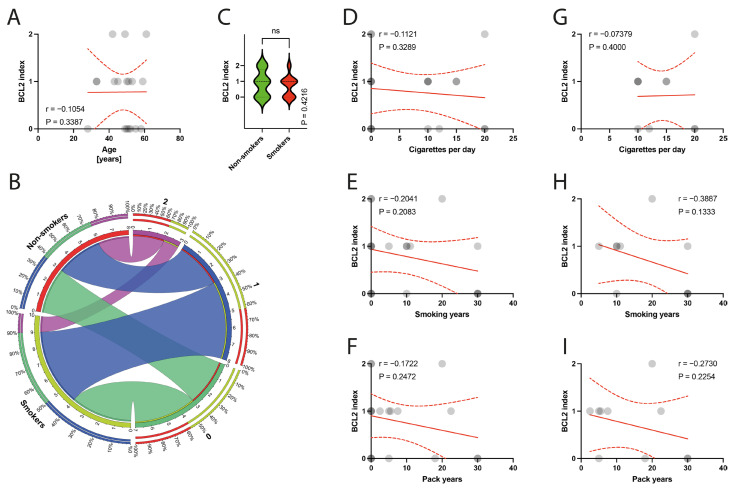
(**A**) Correlation between the BCL2 and the age of patients. (**B**) Comparison of smoking and BCL2 index. (**C**) Comparison between smokers/non-smokers and BCL2 index. (**D**–**F**) Correlation between the BCL2 and smoking years, pack-years index, and cigarettes per day in the total population. (**G**–**I**) Correlation between the BCL2 and smoking years, pack-years index, and cigarettes per day in the smokers population. Colors represent distinct groups of patients, while ribbons connecting various regions of the plot signify relationships between different data elements. Circles represent values; red lines represent simple linear regressions, dotted lines represent 95% confidence intervals. ‘ns’ indicates lack of statistical significance.

**Figure 7:**
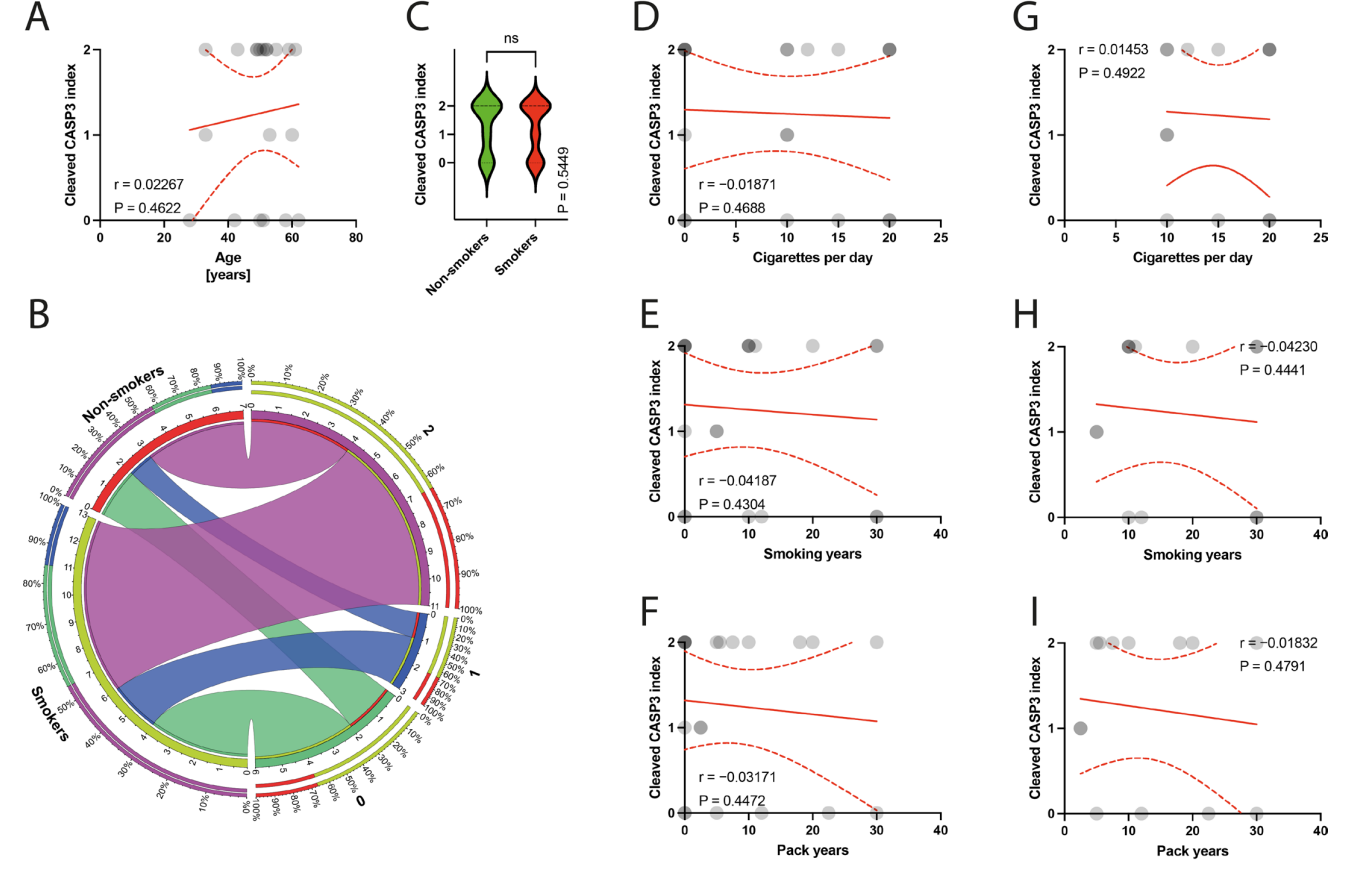
(**A**) Correlation between the cleaved caspase 3 and the age of patients. (**B**) Comparison of smoking and cleaved caspase 3 index. (**C**) Comparison between smokers/non-smokers and cleaved caspase 3 index. (**D**–**F**) Correlation between the cleaved caspase 3 and smoking years, pack-years index, and cigarettes per day in the total population. (**G**–**I**) Correlation between the cleaved caspase 3 and smoking years, pack-years index, and cigarettes per day in the smokers population. Colors represent distinct groups of patients, while ribbons connecting various regions of the plot signify relationships between different data elements. Circles represent values; red lines represent simple linear regressions, dotted lines represent 95% confidence intervals. ‘ns’ indicates lack of statistical significance.

**Figure 8:**
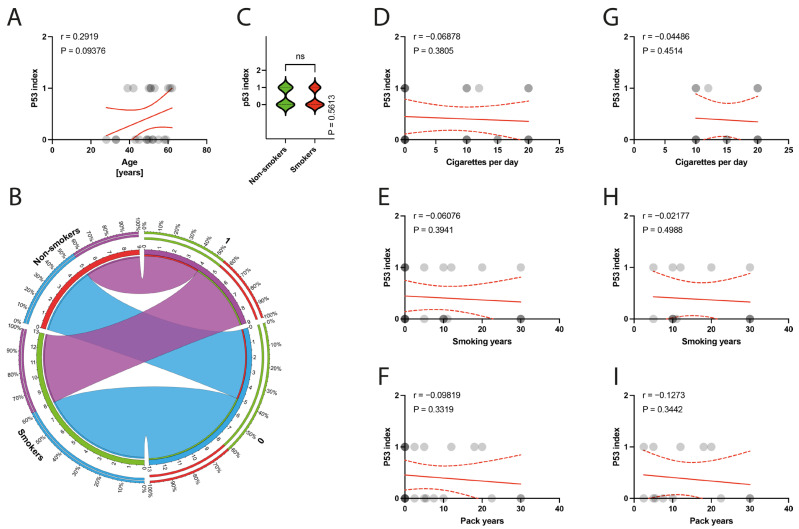
(**A**) Correlation between the p53 index and the age of patients. (**B**) Comparison of smoking and the p53 index. (**C**) Comparison between smokers/non-smokers and the p53 index. (**D**–**F**) Correlation between the p53 index and smoking years, pack-years index, and cigarettes per day in the total population. (**G**–**I**) Correlation between the p53 index and smoking years, pack-years index, and cigarettes per day in the smokers population. Colors represent distinct groups of patients, while ribbons connecting various regions of the plot signify relationships between different data elements. Circles represent values; red lines represent simple linear regressions, dotted lines represent 95% confidence intervals. ‘ns’ indicates lack of statistical significance.

The histological examination of the specimens under a light microscope revealed degeneration of the tendinous tissue in all cases. Additionally, immunohistochemical analysis showed positive expression for BCL2 in 11 patients, cleaved caspase 3 in 14 patients, and p53 in 9 patients. Moderate reactions (1 point) were observed in 8 out of 10 BCL2 reactions, 3 out of 14 cleaved caspase 3 reactions, and in all 9 reactions for p53. Abundant reactions (2 points) were observed in 3 BCL2 reactions, 11 cleaved caspase 3 reactions, and no p53 reactions. There was no correlation between the age of patients and the expression indexes of BCL2, casp3, or p53 (*p* = 0.3387, *p* = 0.4622, *p* = 0.0938, respectively) (Figure 6A, Figure 7A and Figure 8A). Non-smokers and smokers did not show a statistically significant difference in the expression indexes of BCL2, cleaved caspase 3, or p53 (*p* = 0.4216, *p* = 0.5449, *p* = 0.5613, respectively) (Figure 6B,C, Figure 7B,C and Figure 8B,C). Additionally, there was no correlation between smoking indexes and the expression indexes of BCL2, cleaved caspase 3, or p53 (Figure 6D–I, Figure 7D–I and Figure 8D–I). The apoptotic indexes did not correlate with the pre- and postoperative ASES in the entire population (Figures S1A,C, S2A,C and S3A,C), nor in the smoking population (Figures S1B,D, S2B,D and S3B,D). The BCL2 and p53 expression indexes did not correlate with the Snyder system of cuff lesions (Figures S1E,F and S3E,F). However, the cleaved caspase 3 expression index showed a negative correlation with the severity of rotator cuff lesions in the total population according to the Snyder lesion type (*p* = 0.0194) (Figure S2E), but not within the smoking group of patients (*p* = 0.0787) (Figure S2F). Additionally, the apoptotic indexes were not associated with the intensity of pain according to the VAS score in the entire population (Figures S1G, S2G and S3G), nor in isolated smokers (Figures S1H, S2H and S3H).

## 4. Discussion

The most important finding of this study was that despite the detection of apoptotic processes in the LHB tendon, the primary hypothesis had to be rejected, since no statistically significant connection between tobacco smoking, level of apoptosis, and clinical outcomes was demonstrated, except for the cleaved caspase 3 index, which showed an association with the extent of rotator cuff pathology. The secondary hypothesis was confirmed, as it could be shown that smokers had poorer pre- and postoperative results, as well as higher levels of pain. Given the scarcity of evidence, this study contributes by exploring the link between smoking and apoptosis in LHBT tendinopathy.

Programmed cell death plays a crucial role in controlling cell proliferation by regulating tissue morphogenesis and proliferation [11]. There are two main apoptotic pathways: the mitochondrial (intrinsic) and death receptor (extrinsic) pathways [23]. Recent research has shown that these two pathways are interconnected and exhibit reciprocal molecular interaction [24]. The mitochondrial pathway is triggered by various factors (such as oxidative stress, genetic damage, and high cytosolic calcium ion concentrations) that increase mitochondrial membrane permeability [11]. The extrinsic pathway, on the other hand, is activated by transmembrane receptors, such as members of the tumor necrosis factor (TNF) family [25].

The substances present in cigarette smoke have a negative impact on cellular integrity and metabolism, can reduce blood flow to tissues, and can disrupt collagen synthesis, ultimately impairing tendon healing [1,2,4,19]. In 2002, Yuan et al. reported on elevated levels of apoptosis in the supraspinatus tendon in elderly patients, initiating an ongoing discussion about the role of apoptosis in the tendinopathic process of the rotator cuff [26].

Whitin the multifactorial etiology of apoptosis, hypoxia induced by cigarette smoking can activate and enhance apoptotic pathways [11,27]. The hypoxic environment reduces collagen synthesis by tenocytes, impairs the homeostasis of the collagen matrix, and reduces tissue healing. The correlation between rotator cuff pathology and the extent of apoptotic cells may be a reflection of tenocyte adaptation into chondrocyte-like cells, which can survive in low-perfusion environments [27]

Data from previous studies on LHB tendinopathy suggested a correlation between the severity of tendinous tissue degeneration, smoking, clinical outcomes, and the severity of rotator cuff injury [4,8,9]. In this context, the current study could only confirm this association for the relationship between smoking intensity and the severity of rotator cuff pathology classified according to Snyder, but not for the LHB tendon. It is worth noting that LHBT and RCT pathologies can exacerbate each other, and their connection is very strong [28]. Higher stages within the Snyder classification usually reflect more severe rotator cuff tendon pathology, characterized by advanced degeneration of the tissue and a late phase of tendinopathy, often resulting in a tendon tear. Therefore, the extent of the apoptotic process in the neighboring tendons could be reduced due to advanced degeneration. The role of apoptosis in tendinopathy is known to be crucial in the early phase of the pathology, but its role in the late phase is still unclear and inconsistent [11,14]. Our study observed patients with advanced rotator cuff pathology as well as chronic tendinopathy, which could explain the reduced apoptotic population.

In contrast, Chen et al. found a correlation between the age of patients and the extent of the apoptotic process in chronic lateral epicondylitis [29]. They also examined BCL2 19 kDA interacting protein expression, a pro-apoptotic member of the BCL2 family induced by hypoxic conditions as well as inflammation. However, their study included patients who underwent corticosteroid injection, which could bias the biological environment of the tendon specimens [30]. In our study, patients who underwent corticosteroid injection within one year before surgery were excluded to avoid such a bias. Interestingly, Chen et al. noted that in advanced tendon pathology, the apoptotic process was reduced, but still higher than in the control group [29]. This finding is consistent with our findings that the severity of RCT pathology classified by Snyder negatively correlated with cleaved caspase 3.

The extent of apoptosis was observed in several immunohistochemical studies of tendons and ranged from 20% to 34% of cells [20,26,29]. Previous investigations have demonstrated the presence of apoptotic cells in rotator cuff tendons, patellar tendon, Achilles tendon, and extensor carpi brevis tendons [13,29,31]. Specifically, the average apoptotic rate was found to be 28.7% in degenerated ERCB tendons [29], 34% in chronically torn rotator cuff tendons [26], and 24% in supraspinatus tenocytes of patients with stage II subacromial impingement syndrome [32]. In our study, we observed positive immunoreactivity of cleaved caspase 3 with more than 10 cells per specimen in 11 out of 14 patients, while 8 patients did not show any positive reaction. However, the population of tenocytes and apoptotic cells may vary depending on the stage of extracellular matrix transformation during tendinopathic processes [29]. Furthermore, the occurrence of chondrocyte-like transformation may complicate the interpretation of these findings.

In the literature, the presence of programmed cell death is mostly studied in RCT [11,13,19,26,33]. Lundgreen et al. demonstrated an increased number of apoptotic cells not only in partially torn supraspinatus tendons but also in neighboring subscapularis tendon, which could indicate the early onset of a pathological process across the shoulder tendons [33]. Conversely, Benson et al. investigated different stages of rotator cuff tendinopathy and found that apoptosis was only increased in full-thickness tears [27]. Tuoheti et al. also reported increased apoptosis in tendinopathic, non-torn supraspinatus tendons, which supports the presence of apoptosis in the early phases of tendon pathology [32]. Our study also suggests that a lack of apoptotic processes reflects the late stages of LHBT tendinopathy, which is associated with an increased pathology of RCTs. However, Lundgreen et al., in their study on the harmful effects of tobacco smoke on RCTs, showed advanced degeneration and apoptosis in smoking group as compared to healthy patients [19]. The non-smoking population in our study was younger than that in Lundgreen et al.’s study (mean age 44 years vs. mean age 58 years), which may have biased the findings on the presence of apoptotic alterations.

This study include patients into non-smokers group from those, who had never used any nicotine supplement, such as nicotine gum or patch, oral snuff/moist snuff, cigars, or cigarettes. However, the patients called passive smokers were not included. Important fact is, that exposure to environmental tobacco smoke can happen either as inhalation of direct cigarette smoke (second-hand smoke) or its associated residue particles (third-hand smoke), especially when living with a smoker in the same family. It is the reason for approximately 1% of global mortality [34]. It should be accented that exclusion of passive smokers from the non-smokers can be difficult and challenging. Moreover, the type of consumed cigarettes by smokers is an important issue [35]. The present study included patients who smoked conventional cigarettes, however, the impact of smoking may be different in liquid or heating e-cigarettes form.

The main limitation of our study was the small sample size, which may be underpowered to detect certain significant interactions but reflects the exploratory character of the present study. Furthermore, studies focusing on tendinous tissue pathology often have similar patient numbers [19,29,33]. The findings should be also interpreted with caution due to the concurrent presentation of disorders in both LHB and RC tendons, which is a common limitation. Moreover, during the arthroscopic procedure, we were unable to obtain a complete proximal tendon of the biceps. Nevertheless, we attempted to obtain the part that was most exposed to pathological forces. It is important to note that due to the observational study design, there is a limitation in estimating the dose-dependency rate of smoke and histological limitations, reflecting a common limitation in this field. In our study, establishing a control group was not indicated, as obtaining an LHBT specimen from a healthy person may raise ethical concerns. The complexity of LHBT tendinopathy makes it difficult to separate influencing factors such as work or sport, although patients included in the protocol were a non-athletic population and non-heavy workers to minimize risk of distortion of the result, but that may be considered as a study limitation. Finally, three patients were ultimately excluded from the final assessment due to flaws in the processing of the preparation for histological analyses.

## 5. Conclusions

While apoptotic processes in the LHB tendon were observed, no significant association was found between tobacco smoking, the extent of apoptosis, and clinical outcomes. However, expression of the apoptotic marker cleaved caspase 3 correlated with severity of rotator cuff pathology. Furthermore, active smoker status was associated with worse clinical outcomes in terms of pain following LHB tenodesis or tenotomy.

## Data Availability

The data presented in this study are available on request from the corresponding author. The data are not publicly available due to patient research permit.

## Supplementary Materials

The following supporting information can be downloaded at: https://www.mdpi.com/article/10.3390/jcm13030684/s1, Figure S1. (Supplementary). (A) Correlation between the BCL2 and the preoperative ASES—entire population. (B) Correlation between the BCL2 and the preoperative ASES—smoking population. (C) Correlation between the BCL2 and the postoperative ASES—entire population. (D) Correlation between the BCL2 and the preoperative ASES—smoking population. (E) Correlation between the BCL2 and Snyder classification—entire population. (F) Correlation between the BCL2 and Snyder classification—smoking population. (G) Correlation between the BCL2 and VAS—entire population. (H) Correlation between the BCL2 and VAS—smoking population. Figure S2. (Supplementary). (A) Correlation between the cleaved caspase 3 and the preoperative ASES—entire population. (B) Correlation between the cleaved caspase 3 and the preoperative ASES—smoking population. (C) Correlation between the cleaved caspase 3 and the postoperative ASES—entire population. (D) Correlation between the cleaved caspase 3 and the preoperative ASES—smoking population. (E) Correlation between the cleaved caspase 3 and Snyder classification—entire population. (F) Correlation between the cleaved caspase 3 and Snyder classification—smoking population. (G) Correlation between the cleaved caspase 3 and VAS—entire population. (H) Correlation between the cleaved caspase 3 and VAS—smoking population. Figure S3. (Supplementary). (A) Correlation between the p53and the preoperative ASES—entire population. (B) Correlation between the p53and the preoperative ASES—smoking population. (C) Correlation between the p53 and the postoperative ASES—entire population. (D) Correlation between the p53 and the preoperative ASES—smoking population. (E) Correlation between the p53 and Snyder classification—entire population. (F) Correlation between the p53 and Snyder classification—smoking population. (G) Correlation between the p53 and VAS—entire population. (H) Correlation between the p53 and VAS—smoking population.
