# Supplementary material for: The Association of Tobacco Smoking and Level of Apoptosis in the Long Head of the Biceps Chronic Tendinopathy—An Immunohistochemical Study
Source: J Clin Med. 2024 Jan 24;13(3):684. doi: 10.3390/jcm13030684 (PMC10856346; doi:10.3390/jcm13030684)
Supplement: Supplementary file 1 [file jcm-13-00684-s001.zip › jcm-2794541-supplementary.pdf]

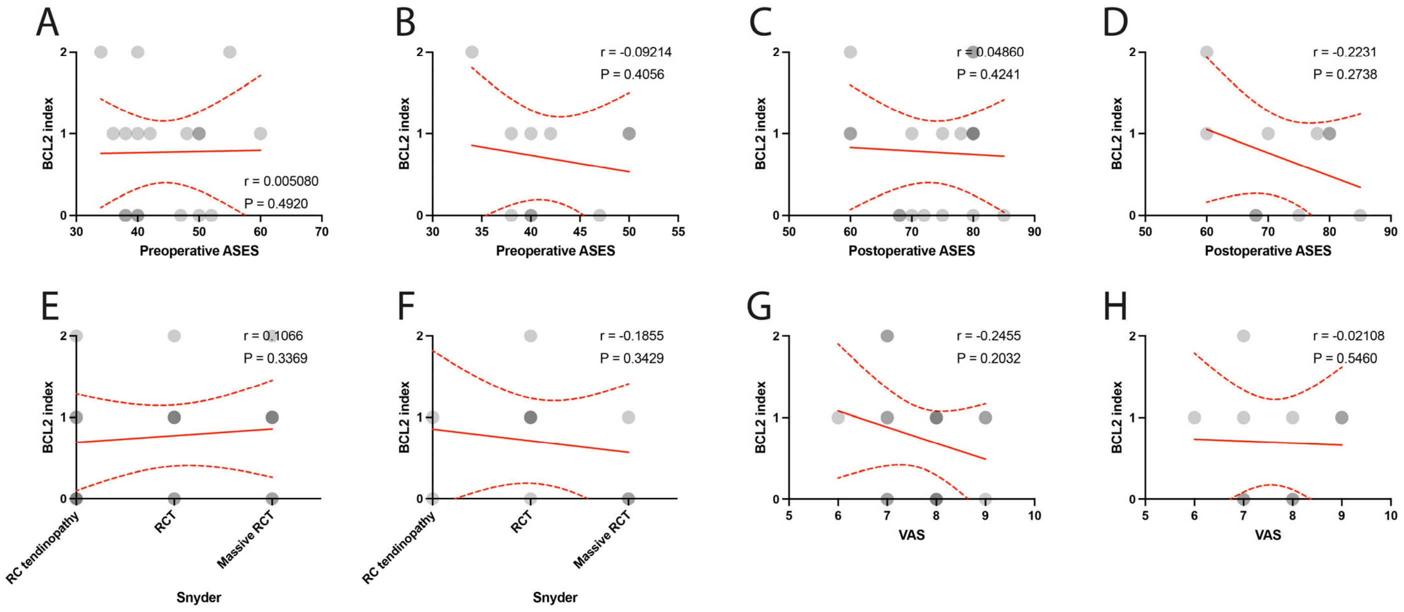

Figure S1. (Supplementary). (A) Correlation between the BCL2 and the preoperative ASES – entire population. (B) Correlation between the BCL2 and the preoperative ASES – smoking population. (C) Correlation between the BCL2 and the postoperative ASES – entire population. (D) Correlation between the BCL2 and the preoperative ASES – smoking population. (E) Correlation between the BCL2 and Snyder classification – entire population. (F) Correlation between the BCL2 and Snyder classification – smoking population. (G) Correlation between the BCL2 and VAS – entire population. (H) Correlation between the BCL2 and VAS – smoking population.

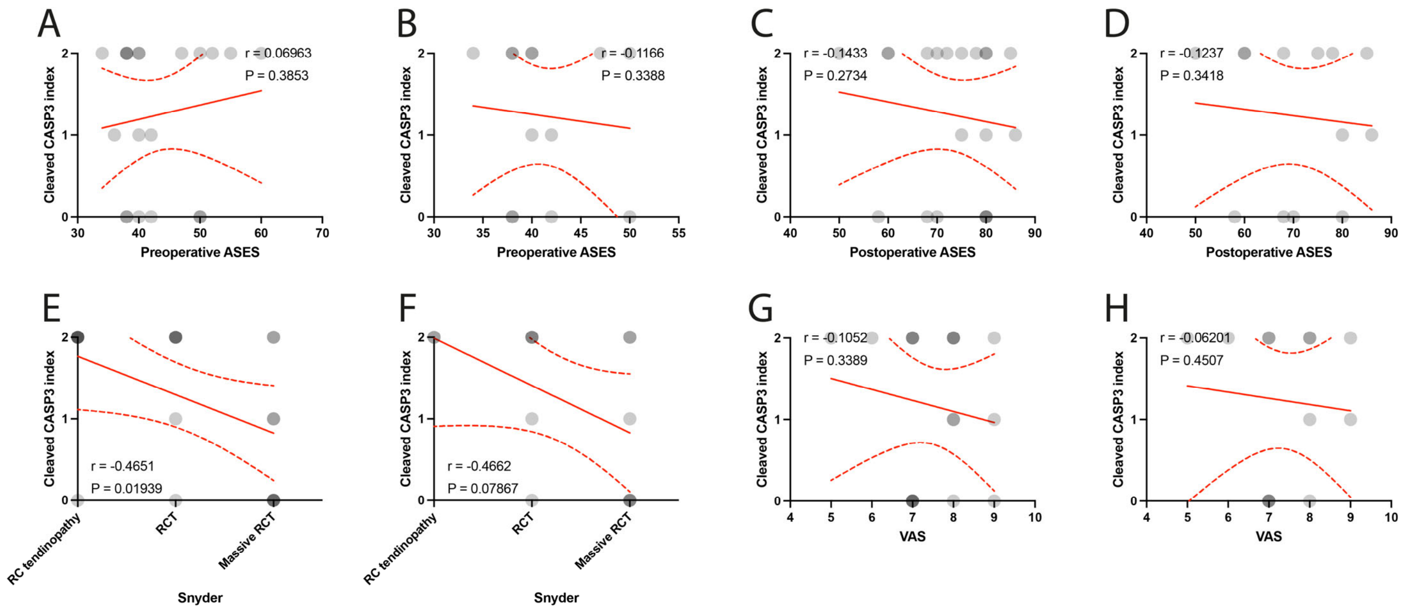

Figure S2. (Supplementary). (A) Correlation between the cleaved caspase 3 and the preoperative ASES – entire population. (B) Correlation between the cleaved caspase 3 and the preoperative ASES – smoking population. (C) Correlation between the cleaved caspase 3 and the postoperative ASES – entire population. (D) Correlation between the cleaved caspase 3 and the preoperative ASES – smoking population. (E) Correlation between the cleaved caspase 3 and Snyder classification – entire population. (F) Correlation between the cleaved caspase 3 and Snyder classification – smoking population. (G) Correlation between the cleaved caspase 3 and VAS – entire population. (H) Correlation between the cleaved caspase 3 and VAS – smoking population.

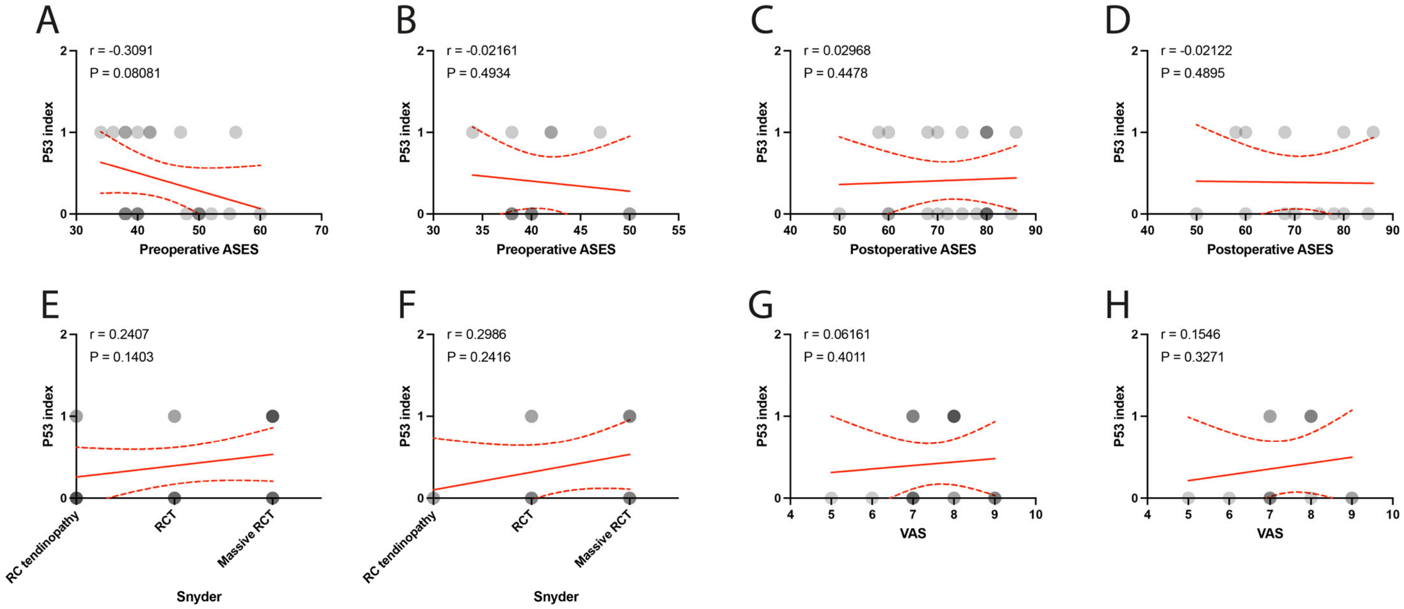

Figure S3. (A) Correlation between the p53and the preoperative ASES – entire population. (B) Correlation between the p53and the preoperative ASES – smoking population. (C) Correlation between the p53 and the postoperative ASES – entire population. (D) Correlation between the p53 and the preoperative ASES – smoking population. (E) Correlation between the p53 and Snyder classification – entire population. (F) Correlation between the p53 and Snyder classification – smoking population. (G) Correlation between the p53 and VAS – entire population. (H) Correlation between the p53 and VAS – smoking population.
